# A Rare Cause of Reversible Renal Hemosiderosis

**DOI:** 10.1155/2015/464059

**Published:** 2015-09-30

**Authors:** Rima Abou Arkoub, Don Wang, Deborah Zimmerman

**Affiliations:** ^1^Division of nephrology, The Ottawa Hospital, Ottawa, ON, Canada K1H 7W9; ^2^Division of Anatomical Pathology, Department of Pathology and Laboratory Medicine, The Ottawa Hospital and Kidney Research Centre of the Ottawa Hospital Research Institute, University of Ottawa, Ottawa, ON, Canada K1H 7W9; ^3^Division of Nephrology, The Ottawa Hospital and University of Ottawa, Ottawa, ON, Canada K1H 7W9

## Abstract

Kidney failure secondary to renal hemosiderosis has been reported in diseases with intravascular hemolysis, like paroxysmal nocturnal hemoglobinuria, and valvular heart diseases. We present here a case of hemosiderin induced acute tubular necrosis secondary to intravascular hemolysis from Clostridium difficile infection with possible role of supratherapeutic INR. We discuss the pathophysiology, causes, and prognosis of acute tubular injury from hemosiderosis.

## 1. Case Report Presentation

A 54-year-old Caucasian man presented to his infectious disease physician in October 2014 with fatigue, nausea, and anorexia for the last 5 days that had been preceded by several episodes of bloody diarrhea of 10 days' duration. He denied abdominal pain, diminished urine output, or macroscopic hematuria. His past medical history was significant for HIV infection for 10 years with the most recent CD4 count of 180; current treatment consisted of Atazanavir, Emtricitabine, Tenofovir, and Ritonavir. He also had a history of chronic hepatitis C infection. He had a previous history of injection drug use that had been complicated by 2 episodes of infective endocarditis requiring a mechanical mitral valve replacement in 1997. Since that time, he had been treated with warfarin anticoagulation, INR 2.5–3.5. He admitted taking additional doses of warfarin when he had migraine headaches as he thought this would improve blood flow. On physical examination, he was afebrile with a supine blood pressure (BP) of 109/65 mmHg but with postural drop to 95/55 mmHg. His JVP was flat with no evidence of lower extremity edema. He did not have a skin rash or detectable heart murmur. The remainder of his examination was otherwise unremarkable.

His laboratory data on admission are outlined in Table 1. His urinalysis was significant for 5–30 RBC/HPF and hyaline casts; no heme granular or red blood cell casts were seen. His urine albumin : creatinine ratio was 217 g/mol Cr. His creatinine which was 1.4 mg/dL in July 2014 had increased to 19.9 mg/dL. Abdominal ultrasonography showed normal-sized kidneys with slightly increased right renal parenchymal echogenicity.

**Figure 1:**
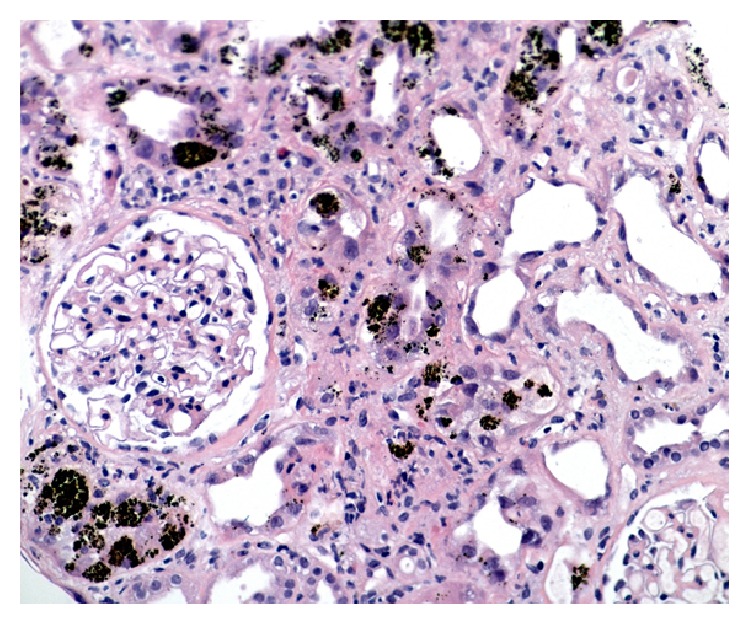
H&E stain showing extensive iron deposits in the tubules and glomerulus spared from deposition (H&E, ×300).

**Figure 2:**
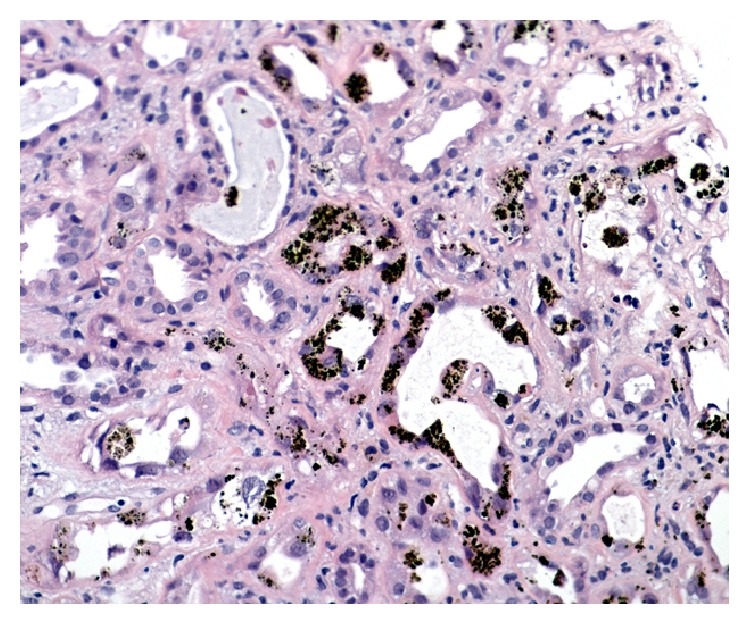
H&E stain showing severe acute tubular necrosis (ATN) caused by extensive iron depositions in the tubules (H&E, ×300).

**Figure 3:**
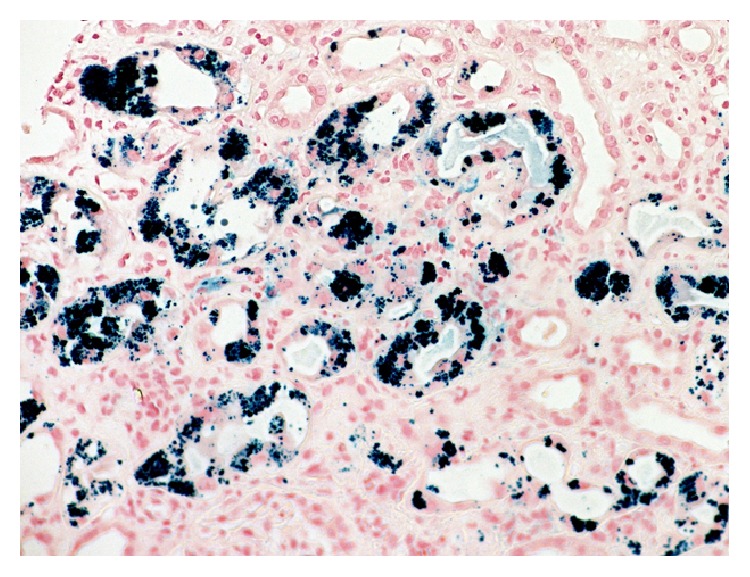
Prussian blue stain showing strong diffuse positivity in the tubules as coarse blue granules (Prussian blue, ×300).

**Figure 4:**
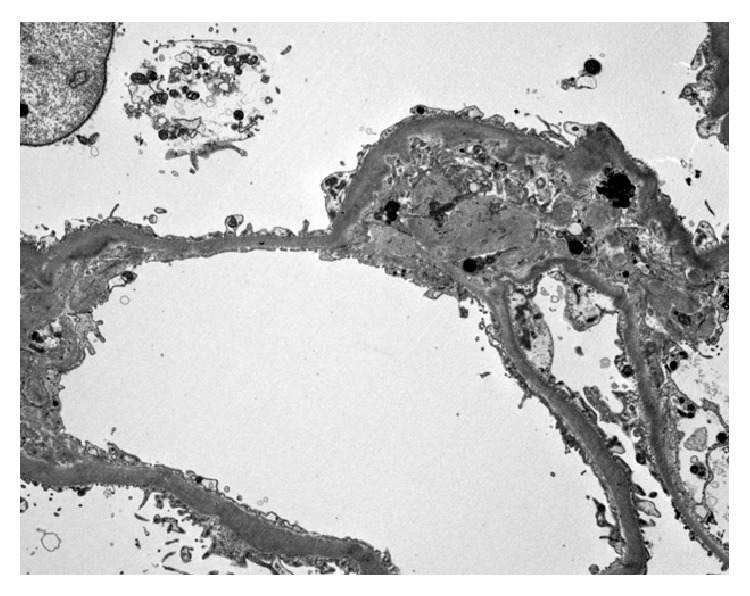
Electron microscopy shows segmental effacement of foot process with iron deposits (dark granules) (electron microscopy magnification ×8000).

After fluid resuscitation failed to improve his acute kidney injury (AKI), he underwent hemodialysis for metabolic acidosis. Other investigations included (1) anemia work-up for a declining hemoglobin [11.1 g/dL to 6.6 g/dL after fluid resuscitation without evidence of bleeding or falling platelet count (137 × 10^9^/L)] included blood film review which showed polychromasia without schistocytes, LDH 153 (N: 100–205 units), haptoglobin < 0.08 (N: 0.3–2.0 units), total bilirubin of 43 (N: 3–17 units), direct bilirubin of 6 (N: 2–9 units), iron percent saturation 86 (N: 20–50), and ferritin 314 (N: 24–336), making TTP and HUS unlikely with the stable platelet count and absence of schistocytes on blood smear. Further investigations showed (1) a positive random urine for hemosiderin and (2) renal biopsy that showed severe acute tubular necrosis (ATN) caused by extensive renal hemosiderosis with extensive iron depositions in the tubules; the glomeruli were spared from deposition and damage (Figures 1 and 2). There was focal interstitial chronic inflammation, mild interstitial fibrosis, mild tubular atrophy, and mild-to-moderate arterial atherosclerosis. Immunofluorescent staining: IgG: Negative, IgA: negative, IgM: equivocal, complement (C3): equivocal, C1q: negative, albumin: negative, and Kappa/Lambda: negative. Special stains for iron show strong diffuse positivity in the tubules (Figure 3). Electron microscopy showed segmental effacement of foot process with no evidence of electron dense deposits (Figure 4).

(3) Clostridium difficile toxin B was detected by PCR, (4) normal genetic testing for HFE (human hemochromatosis protein gene) mutation excluding hemochromatosis, negative flow cytometry for PNH (paroxysmal nocturnal hemoglobinuria), a normal glucose-6-phosphate dehydrogenase screen (excluding hemolysis secondary to G6PD deficiency anemia), and (5) transesophageal echocardiogram which showed that the prosthetic mitral valve is functioning well without evidence of paravalvular leak or mitral valve regurgitation

The patient stabilized in hospital with treatment of his AKI and Clostridium difficile infection. He remained on 3-times-per-week hemodialysis for ten weeks when his AKI recovered and his central venous catheter was removed.

## 2. Discussion

We report a case of acute hemolysis and nonoliguric AKI secondary to hemosiderin induced tubular necrosis. Upon hemolysis of red blood cells, hemoglobin alpha-beta dimers are released that, if unbound to haptoglobin, are filtered by the glomerulus and appear in the urine as hemoglobinuria. The hemoglobin dimers are taken up by renal proximal tubular cells and degraded and the free chelatable iron is stored as hemosiderin in the lysosomes [1, 2]. Under normal conditions, modest hemosiderin deposition is only mildly toxic to the kidney. However, with hemolysis in the presence of concentrated urine secondary to extracellular fluid volume depletion, hemosiderin can induce hemoglobinuria-associated acute renal failure [1]. Cytotoxic effects of large amounts of heme result from its lipophilic, oxidant, proinflammatory, and apoptotic effects.

Studies in an experimental model of heme protein-induced kidney injury show that mitochondria in particular are vulnerable to heme-mediated damage [3].

Hemosiderin deposition in kidney tubular cells has been reported in different types of hemolytic anemia including autoimmune hemolytic anemia and PNH [1, 4, 5] and sickle cell anemia [6, 7] and after cardiac valve replacement with residual valvular regurgitation or perivalvular leak [8]. In hemochromatosis, hemosiderin accumulation in tubular epithelial cells has also been reported [9]. ATN secondary to highly active antiretroviral therapy (HAART) including Tenofovir has been reported in HIV-infected patients. Moreover approximately 70% of the published cases of Tenofovir-induced nephrotoxic effects are observed with concomitant use of low-dose Ritonavir, the combination used by our patient [10]. However iron deposition in tubular cells causing ATN has not been reported with these medications making this an unlikely cause of this patient's renal failure.

All of these possibilities were considered in our differential diagnosis but all of them were subsequently excluded. Other potential etiologies included hemolysis secondary to Clostridium difficile infection or overanticoagulation with warfarin.

Several infections have also been associated with hemolysis and hemosiderin in the urine. Since 1990, 50 patients with Clostridium perfringens septicemia with hemolysis have been reported [11]; renal failure was reported in some but there was no mention of biopsy proven renal hemosiderosis. There are also case reports of hemolysis and severe jaundice in G-6-PD-deficient neonates with Clostridium difficile infection [12]. However, renal involvement and acute kidney injury did not appear to be part of the presentation in infants.

With respect to the potential role of warfarin and the supratherapeutic INR in our patient, Brodsky et al. examined renal biopsy specimens from 9 patients with AKI and an abnormal international normalized ratio (mean 4.4 ± 0.7 IU). There was evidence of acute tubular injury and glomerular hemorrhage: red blood cells in Bowman space with numerous occlusive RBC casts in the tubules, none with hemosiderin deposition [13]. However in one other case report, a kidney biopsy from a patient with IgA nephropathy, macroscopic hematuria, AKI, and INR of 6.2 IU showed extensive interstitial and intratubular red blood cell extravasation and interstitial hemosiderin deposits [14].

In summary, our patient appears to have developed AKI secondary to hemosiderin induced acute tubular necrosis perhaps from a combination of extracellular fluid volume depletion, elevated INR, and Clostridium difficile infection.

## Figures and Tables

**Table 1:** Basic laboratory data on admission.

Creatinine	19.9	mg/dL
Urea	95.2	mg/dL
Potassium	3.7	mEq/L
Sodium	124	mEq/L
CO_2_	10	mEq/L
Chloride	95	mEq/L
INR	5.9	
Hemoglobin	11.1	g/dL
WBC	7.8	×10^9^/L
MCV	97	fL
Platelets	164	×10^9^/L
